# Feasibility of perampanel monotherapy for seizure management during awake craniotomy for glioma

**DOI:** 10.1007/s11060-026-05580-w

**Published:** 2026-04-24

**Authors:** Yuta Koketsu, Shoichi Deguchi, Fumiharu Ohka, Kosuke Aoki, Yoshiki Shiba, Yuhei Takido, Shigeaki Nawa, Takehito Sato, Koichi Akiyama, Ryuta Saito

**Affiliations:** 1 Department of Neurosurgery, Nagoya University School of Medicine, Nagoya, Aichi Japan; 2 Department of Anesthesiology, Nagoya University School of Medicine, Nagoya, Aichi Japan

**Keywords:** Perampanel, Monotherapy, Glioma, Awake surgery, Intraoperative seizure outcome, Oncology

## Abstract

**Purpose:**

This study evaluated the feasibility of perampanel (PER) monotherapy for perioperative seizure management in patients undergoing awake craniotomy (AC) for glioma, with emphasis on preventing intraoperative intractable seizures.

**Methods:**

A retrospective analysis was conducted on patients who underwent AC at our institution between December 2024 and December 2025 and received PER monotherapy. PER was administered preoperatively at 2–4 mg. On the day of surgery, an additional 2 mg of PER was given intravenously at the start of the procedure and another 2 mg upon completion.

**Results:**

Of 29 patients who underwent AC, five were excluded (four on multiple antiseizure medications and one who declined PER treatment), leaving 24 for analysis. Intraoperative seizures occurred in 3 patients (12.5%), all focal and controlled with cold saline irrigation. No intractable or generalized seizures occurred, and no awake procedures failed. Two patients had preoperative epilepsy, whereas one had no prior seizure history. Early postoperative seizures developed in 2 patients (8.3%), and complete seizure control was achieved in all patients by 3 months postoperatively. Treatment-related adverse effects occurred in one patient (4.2%) who developed a skin rash and discontinued PER. Among the 22 patients without early postoperative seizures, antiseizure medications were successfully discontinued within 6 months in 9 patients.

**Conclusion:**

In this cohort, PER provided perioperative seizure control with a very low incidence of adverse effects. These preliminary findings support the feasibility of PER monotherapy and provide a rationale for prospective evaluation.

**Clinical trial number:**

Not applicable.

## Introduction

Awake craniotomy (AC) enables maximal safe resection of gliomas located in eloquent brain regions [1]. It allows real-time cortical and subcortical mapping, thereby facilitating more extensive tumor removal while minimizing the risk of permanent neurological or language deficits [2]. AC is an effective strategy to preserve neurological function during tumor removal, and has been associated with a greater extent of resection, lower rates of new neurological deficits, and better postoperative quality of life [3–5]. 

Intraoperative seizures (IOS) are among the most significant complications during AC [6]. IOS can interrupt mapping and may require conversion to general anesthesia. They often occur during cortical stimulation for functional mapping [7]. The incidence of IOS varies widely, ranging from 3.4 to 24% [8]. 

Seizure prevention during AC requires special consideration because IOS following craniotomy can cause increased intracranial pressure, neurological deterioration, cerebral hemorrhage, or hypoxia. Therefore, seizure management in AC should be regarded as a distinct clinical concern [9], even though recent recommendations advise against routine use of prophylactic antiseizure medication (ASM) in patients with newly diagnosed brain tumors [10]. 

Our group reported that a combination of levetiracetam (LEV) and perampanel (PER) administered before AC reduces IOS compared with LEV alone [11]. However, another recent study suggested that single-agent prophylaxis may be preferable to combination therapy when ASM is used [2]. This is because the superiority of combination therapy over monotherapy has not been clearly demonstrated, while combination therapy may increase adverse events and drug interactions. PER monotherapy before AC has been reported in a single case report [12], but its clinical effectiveness for prevention of IOS remains unclear. The present study investigated PER as a single prophylactic agent for seizure prevention during AC and evaluates its clinical utility.

## Methods

### Ethical considerations

The study protocol was approved by the Institutional Review Board of Nagoya University Hospital (approval number: 2025 − 0505). Written informed consent was obtained from all patients.

### Study design and patient population

**Fig. 1:**
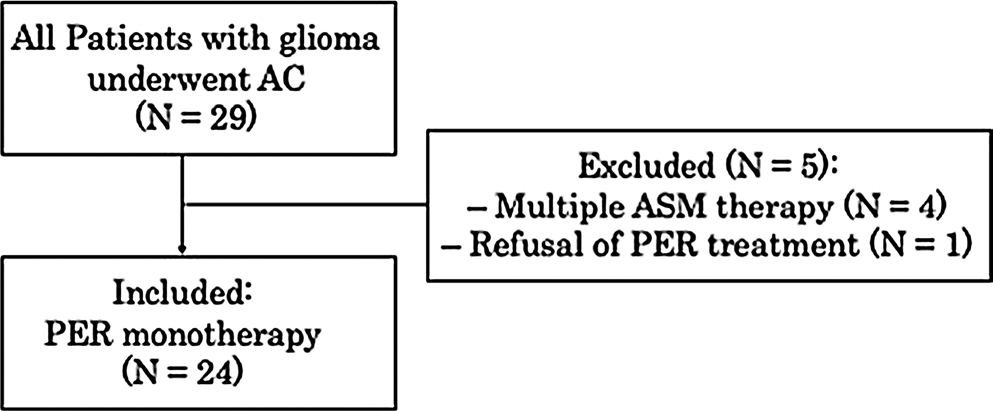
Patient selection for the awake craniotomy glioma cohort

This retrospective cohort study included 29 consecutive patients with supratentorial glioma who underwent AC with intraoperative direct electrical stimulation mapping at the Department of Neurosurgery, Nagoya University Hospital, between December 2024 and December 2025. Patients enrolled in the ongoing prospective multicenter GRAMPAS trial, which includes our institution and evaluates perioperative prophylactic administration of PER 2 mg for supratentorial tumors, were not included in this retrospective analysis [13]. Five patients were excluded: four due to preoperative status epilepticus requiring treatment with multiple ASMs, and one because the patient declined PER treatment (Fig. 1).

Clinical data collected included patient demographics (age, sex), initial Karnofsky Performance Scale (KPS) score, tumor characteristics, extent of resection (EOR), isocitrate dehydrogenase (IDH) 1/2 mutation status, 1p/19q status, O‑6‑Methylguanine‑deoxyribonucleic acid (DNA) methyltransferase (MGMT) promotor methylation status, dose of PER, direct cortical stimulation intensity, number of cortical stimulations, follow-up duration, and seizure outcomes. Postoperative early seizures were defined as those occurring within a week after surgery, and postoperative late seizures as those occurring up to 3 months postoperatively in accordance with our previous study [11]. In addition, treatment-related adverse events were evaluated until the last follow-up visit.

### PER administration

**Fig. 2:**
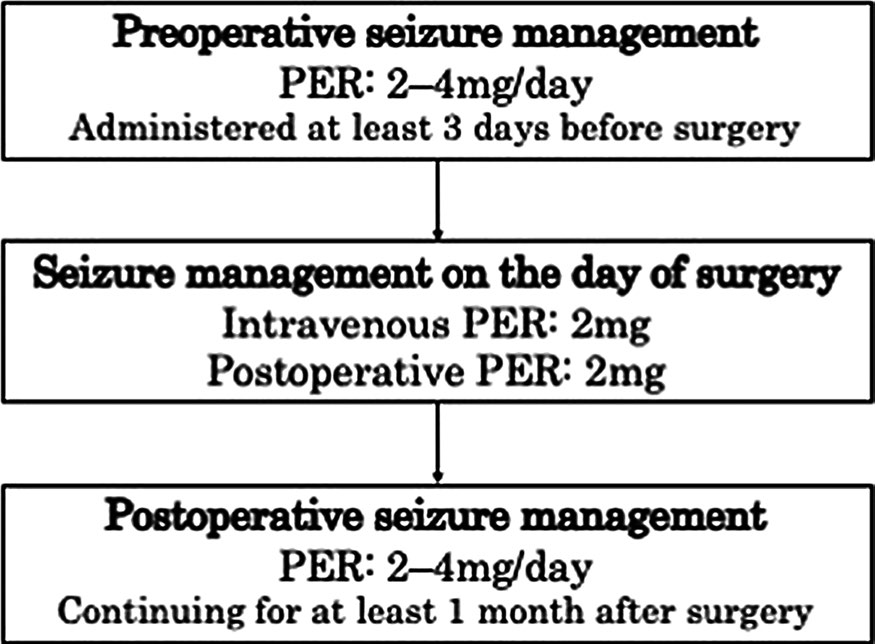
Perioperative perampanel monotherapy regimen. All patients received oral perampanel 2 mg at least 3 days before surgery, with escalation to 4 mg as determined by the treating physician, particularly in those with a longer preoperative interval or preoperative seizures. On the day of surgery, perampanel 2 mg was given intravenously after induction of general anesthesia and another 2 mg after return to the ward, and the preoperative antiseizure medication regimen was then resumed and continued for at least 1 month unless intractable seizures occurred

PER monotherapy was initiated in all patients at least 3 days before surgery at an initial dose of 2 mg, regardless of prior seizure history. When the interval before surgery exceeded 2 weeks, the treating physician determined whether to increase the dose to 4 mg based on each patient’s clinical condition, particularly the history of preoperative seizures. When the preoperative interval was shorter, patients underwent surgery while maintained at 2 mg. On the day of surgery, intravenous PER (2 mg) was administered after induction of general anesthesia, and an additional 2 mg was given upon return to the ward. Following the surgery, the ASM regimen was resumed immediately according to the preoperative regimen. Postoperative antiepileptic management was continued for at least 1 month (Fig. 2).

### Surgical procedure

All ACs with direct brain stimulation were performed using the asleep–awake–asleep technique, as previously described [11, 14–18]. Anesthesia was induced with total intravenous anesthesia (TIVA) using propofol and remifentanil, and the airway was secured with an i-gel laryngeal mask (Intersurgical, Inc.) [19]. A scalp nerve block was administered by experienced anesthesiologists prior to head frame fixation [20]. Bilateral supraorbital, auriculotemporal, and greater and lesser occipital nerves were anesthetized using ropivacaine (0.375%) with epinephrine. The head pin insertion sites and planned incision sites were infiltrated with ropivacaine. The dura mater was infiltrated with ropivacaine without epinephrine after craniotomy. After opening the dura, TIVA was discontinued to allow the patient to awaken spontaneously. Following completion of the awake phase, general anesthesia was resumed, and the wound was closed in the standard manner.

Intraoperative functional mapping was performed as previously reported [11, 14–18]. A bone flap was fashioned to expose the cortex, and tumor margins were identified using neuronavigation. Direct cortical stimulation was delivered using a bipolar stimulator (Unique Medical) with biphasic current (60 Hz, 0.5 ms). Stimulation intensity was gradually increased from 1.0 mA up to a maximum of 4.0 mA until a reproducible response was obtained, and the determined threshold was used for both cortical and subcortical mapping.

Continuous electrocorticography (ECoG) was used to monitor afterdischarges during stimulation and tumor resection. Language mapping was performed using counting and picture‑naming tasks combined with direct electrical stimulation. Speech therapists assessed patients for language disturbances during mapping. Tumor resection was continued until both cortical and subcortical functional boundaries were delineated.

### Intraoperative seizure management

If clinical seizures or stimulation-induced epileptiform discharges were detected on ECoG during awake mapping, stimulation was immediately suspended and the cortical surface was irrigated with cold saline (0.9% NaCl) until the activity ceased. Tumor resection was resumed once the patient was able to perform language or motor tasks. Seizures controlled by cold saline were defined as controllable seizures, whereas persistent seizures requiring termination of the awake procedure and conversion to general anesthesia were defined as intractable seizures.

### Imaging and molecular analysis

Preoperative MRI was obtained using a 3.0-T machine (Trio, Siemens) and included 3D T1-weighted imaging, conventional T1- and T2-weighted sequences, and diffusion-weighted imaging. Postoperative T1- and T2-weighted MRI was performed approximately 3 days and again 3 months after surgery to assess the EOR. Volumetric tumor analyses were conducted using Brainlab Viewer software (version 5.4; Brainlab AG, Munich, Germany). Tumor volumes were measured on contrast-enhanced T1-weighted images for enhancing tumors and on Fluid‑Attenuated Inversion Recovery (FLAIR) images for non-enhancing tumors. EOR was calculated as (preoperative tumor volume − postoperative tumor volume) / preoperative tumor volume [21]. 

Direct sequencing of IDH1/2 was performed as previously described [22, 23]. Fragments covering the catalytic domain of IDH1 (including codon 132) and IDH2 (including codon 172) were amplified and sequenced. Copy number status of 1p and 19q was assessed using multiplex ligation-dependent probe amplification (MLPA) [24], performed at a commercial laboratory (FASMAC, Kanagawa, Japan). Codeletion of 1p/19q was defined as concordant signal loss across both loci below the predefined cutoff. In grade 4 tumors, MGMT promoter methylation status was determined by methylation‑specific PCR following bisulfite treatment of genomic DNA [25]. 

### Literature search

A focused, non‑systematic search of the PubMed database was performed to identify clinical series of AC for glioma conducted under routine ASM use. From the selected reports, we extracted publication year, number of patients, incidence of IOS, rate of AC failure, and details of the ASM regimen, and summarized these data in a comparative table.

### Statistical analysis

Statistical analyses were performed using EZR (version 1.68; Saitama Medical Center, Jichi Medical University), a graphical user interface for R [26]. Comparisons between groups were conducted using Fisher’s exact test. Proportions are presented with two-sided 95% confidence intervals (CI), which were calculated using the Clopper–Pearson method. Statistical significance was defined as *p* < 0.05.

### Data availability

The datasets generated and analysed during the current study are not publicly available due to patient privacy and institutional regulations, but are available from the corresponding author on reasonable request and with approval from the institutional review board.

## Results

### Patient population

A total of 24 patients (13 males and 11 females) who received PER monotherapy prior to AC were included in the final analysis. Baseline characteristics are summarized in Table 1. 14 patients (58.3%) underwent surgery with PER at 2 mg and 10 (41.7%) with 4 mg. The median age at surgery was 49 years (range, 22–72), and the median KPS score was 100. Fourteen patients (58.3%) had left-sided tumors. Tumor locations included the frontal lobe in 13 patients (54.2%), the temporal lobe in 5 patients (20.8%), the parietal lobe in 4 patients (16.7%), and the insular region in 2 patients (8.3%). Motor-related regions were involved in 6 patients (25.0%). The median preoperative tumor volume was 19.8 cm^3^ (range, 0.2–166.2). According to the World Health Organization classification, 12 patients (50.0%) had grade 2 tumors, 2 (8.3%) had grade 3 tumors, and 10 (41.7%) had grade 4 tumors. IDH1/2 mutation analysis showed that 12 patients (50.0%) were wild type and 12 (50.0%) had mutations. Regarding 1p/19q status, 6 patients (25.0%) had codeleted tumors and 18 (75.0%) had non-codeleted tumors. Among the patients with grade 4 tumors, MGMT promotor methylation status was methylated in 4 (40.0%), and unmethylated in 6 (60.0%). The median stimulation intensity during cortical mapping was 2.0 mA (range, 1.5–4.0), and the median number of cortical stimulations was 54 (range, 22–196). The median intraoperative awakening time was 1.73 h (range, 0.83–2.58). The median EOR was 100% (range, 70–100%). The median follow-up duration was 12 months (range, 3–15).

**Table 1:** Clinical characteristics of all patients who underwent awake craniotomy for glioma

Parameter	No. of patients (%)	Parameter	No. of patients (%)
Age (years)		WHO diagnosis	
Median	49	Grade 2	12 (50.0)
Range	22–72	Oligodendroglioma, IDH-mut and 1p/19-codeleted	5 (20.8)
Sex			
Male	13 (54.2)	Astrocytoma, IDH-mut	6 (25.0)
Female	11 (45.8)	Diffuse glioma, IDH-wildtype, NEC	1 (4.2)
Initial KPS score		Grade 3	2 (8.3)
Median	100	Oligodendroglioma, IDH-mut and 1p/19-codeleted	1 (4.2)
Range	70–100		
Side of lesion		Diffuse glioma, IDH-wildtype, NEC	1 (4.2)
Lt	14 (58.3)	Grade 4	10 (41.7)
Rt	10 (41.7)	Glioblastoma, IDH-wild type	10 (41.7)
Tumor location		-MGMT methylated	4 (40.0)
Frontal	13 (54.2)	-MGMT unmethylated	6 (60.0)
Insular	2 (8.3)	Stimulation current intensity (mA)	
Temporal	5 (20.8)	Median	2.0
Parietal	4 (16.7)	Range	1.5–4.0
Involvement of motor-related regions		Number of cortical stimulations	
Positive	6 (25.0)	Median	54
Negative	18 (75.0)	Range	22–196
Tumor volume (cm^3^)		Intraoperative awakening time (hours)	
Median	19.8	Median	1.73
Range	0.2–166.2	Range	0.83–2.58
IDH1/2 mutation status		Final EOR	
Wild type	12 (50.0)	Median	100
Mut	12 (50.0)	Range	70–100
PER dose per day		Duration of follow-up (months)	
2 mg	14 (58.3)	Median	12
4 mg	10 (41.7)	Range	3–15

Abbreviations: EOR, extent of resection; IDH, isocitrate dehydrogenase; KPS, Karnofsky Performance Status; Lt, left; MGMT, O‑6‑Methylguanine‑DNA methyltransferase; mut, mutant; NEC, not elsewhere classified; PER, perampanel; Rt, right; WHO, World Health Organization

### Pre- and postoperative seizure outcomes

Pre- and postoperative seizure outcomes are summarized in Table 2. Ten patients (41.7%) had a history of preoperative seizures, all of which were well controlled before surgery. Among them, 4 received PER at 2 mg and 6 received 4 mg, whereas among the 14 patients without a preoperative seizure history, 10 received 2 mg and 4 received 4 mg. One of the patients with preoperative epilepsy treated with LEV 1000 mg was switched to PER 2 mg three days before surgery, and the remaining 9 patients had received PER monotherapy.

**Table 2:** Perioperative seizure outcomes in patients receiving perampanel monotherapy during awake craniotomy

Parameter	No. of patients (%)
Preoperative seizure history	
Present	10 (41.7)
Absent	14 (58.3)
Intraoperative seizures	
Controllable seizures	3 (12.5)
Intractable seizures	0 (0.0)
Total	3 (12.5)
Postoperative early seizures	
Present	2 (8.3)
Absent	22 (91.7)
Postoperative late seizures	
Present	0 (0.0)
Absent	24 (100.0)

Early postoperative seizures occurred in 2 patients (8.3%; 95% CI, 1.0–27.0%) within a week after surgery, both of whom were receiving PER 2 mg. All seizures were focal onset. The remaining 22 patients (91.7%) remained seizure-free on the same ASM regimen. At 3 months postoperatively, all patients were seizure-free. Among the 22 patients without early postoperative seizures, ASM was discontinued in 9 patients within 6 months postoperatively (range, 1–6) following shared decision-making. No seizures occurred after ASM discontinuation in any patient.

### Intraoperative seizures

IOS occurred in 3 patients (12.5%; 95% CI, 2.7–32.4%). All were focal-onset seizures triggered by cortical stimulation and resolved with cold saline irrigation. No patient developed intractable seizures or required termination of the awake procedure.

Among the 3 patients with IOS, 2 had preoperative epilepsy and 1 had no prior seizure history. Among the 2 patients with preoperative epilepsy, 1 patient harbored a tumor involving a motor-related region. The patient without preoperative epilepsy experienced early postoperative seizures. In patients with preoperative epilepsy, IOS occurred in 1 of 4 (25.0%) receiving 2 mg of PER and in 1 of 6 (16.7%) receiving 4 mg. In contrast, among patients without a prior seizure history, IOS occurred in 1 of 10 patients (10.0%) receiving 2 mg of PER, whereas no IOS was observed in the 4 patients receiving 4 mg of PER. In both IOS cases that occurred under 2 mg of PER, the drug had been initiated 1 week before surgery.

### Adverse effects

Adverse effects related to treatment occurred in one patient (4.2%; 95% CI, 0.1–21.1%). This patient developed a skin rash while receiving PER 2 mg and discontinued the drug 4 months after surgery. No seizures occurred thereafter. No patients reported irritability, aggression, sleepiness, fatigue, or dizziness.

### Comparison with previous series

Results were compared with previous AC series for glioma using routine perioperative ASM regimens, and the findings are summarized in Table 3 [2, 11, 27, 28]. 

**Table 3:** Outcomes of awake craniotomy for glioma under routine antiseizure medication in previous studies and in the present series

Study	Year	*N*	IOS rate	AC failure	ASM
Wang YC, et al.	2019	41	7.3%	0%	LEV or VPA 1000 mg; add-on AED if needed
Abecassis ZA, et al.	2020	229	15.3%	0.9%	Single-dose LEV 500–1000 mg + Baseline regimen
Motomura K, et al.	2021	50	34.0%	2.0%	LEV 1000–3000 mg
Motomura K, et al.	2021	28	7.1%	0%	LEV 1000–3000 mg + PER 2–4 mg
Pour-Rashidi A, et al.	2025	29	27.6%	13.8%	LEV 1000 mg
Pour-Rashidi A, et al.	2025	26	11.5%	3.8%	LEV 1000 mg + FOS, adjusted by serum level
Present study	2026	24	12.5%	0%	PER 2–4 mg

Abbreviations: AC, awake craniotomy; AED, antiepileptic drug; ASM, antiseizure medication; FOS, fosphenytoin; IOS, intraoperative seizure; LEV, levetiracetam; PER, perampanel; VPA, valproic acid

## Discussion

### Feasibility of PER monotherapy

We evaluated the feasibility of PER monotherapy for IOS control during AC. IOS occurred in 3 of 24 patients (12.5%), all focal-onset and controllable with cold saline irrigation, without secondary generalization or conversion to general anesthesia. This IOS rate and absence of AC failure were comparable to previous AC series under routine ASM use [2, 11, 27, 28]. There was no statistically significant difference in IOS incidence compared with our previous LEV–PER combination therapy cohort (7.1%) (Fisher’s exact test, *p* = 0.647) [11]. However, it should be noted that patient characteristics—particularly the preoperative seizure status—differed between the two cohorts. More specifically, 35.7% of patients in the prior LEV–PER cohort had uncontrolled preoperative seizures, defined as class II–VI in the International League Against Epilepsy (ILAE) classification [29, 30]. In contrast, all patients with preoperative epilepsy in the present cohort exhibited well‑controlled seizures, which may be due to the exclusion of cases receiving multiple ASMs preoperatively. In addition, the proportion of WHO 2021 grade 4 gliomas in the present cohort (41.7%) was higher than that of WHO 2016 grade Ⅳ gliomas in the prior cohort (20–25%), reflecting differences in baseline clinical and tumor characteristics between the two cohorts.

### Intraoperative seizure risk profile

IOS in this study mainly occurred in patients with clinical and anatomical risk factors previously associated with IOS. Prior studies have reported that preoperative seizures, frontal lobe or motor-related lesions, and preoperative ASM use are associated with increased risk of IOS during awake mapping [11, 31]. Among the 3 patients with IOS, 2 had preoperative epilepsy, including 1 with a frontal tumor involving a motor-related region. The third patient had no prior seizure history but developed early postoperative seizure.

Although the number of IOS cases was limited, most events occurred in patients with these risk profiles. These findings suggest that baseline patient and tumor characteristics strongly influence IOS risk. Patients with such risk factors may require closer perioperative monitoring, including careful stimulation and continuous EEG monitoring for after-discharges [32]. 

### Optimal dose of PER

PER is a selective antagonist of the α-amino-3-hydroxy-5-methyl-4-isoxazole-propionic acid receptor and is clinically approved for the treatment of focal–onset and generalized tonic–clonic seizures [33], with a long half–life of approximately 105 h [34, 35]. Although guidelines recommend dose escalation from an initial 2 mg to a maintenance dose of 4–10 mg, previous studies suggest that even 2 mg may be effective in brain tumor–related epilepsy and in perioperative prophylactic settings [36]. We therefore evaluated the optimal PER dose for perioperative seizure prophylaxis during AC.

In our cohort, IOS occurred in 2 of 14 patients (14.3%) receiving PER 2 mg and in 1 of 10 patients (10.0%) receiving 4 mg, with no statistically significant difference between the two groups (Fisher’s exact test, *p* = 1.00). Given the small sample size, the present data may be underpowered to draw firm conclusions about the preventive effect of each dose on IOS.

Regarding the postoperative course, two patients receiving PER 2 mg—one without and one with a history of preoperative seizures—experienced focal seizures in the early postoperative phase, and seizure control was subsequently achieved with continuation of the same ASM regimen.

Overall, these findings suggest that PER doses of 2–4 mg are feasible for preventing IOS. However, the optimal dose for preventing IOS may differ from that required for postoperative seizure prophylaxis because of differences in seizure mechanisms and perioperative risk factors.

### Safety and postoperative management

Treatment–related adverse effects occurred in 1 patient (4.2%) who developed a skin rash and discontinued PER 4 months after surgery. No seizures occurred thereafter. No patients reported sleepiness, fatigue, or dizziness. In a previous study of LEV–PER combination therapy, these symptoms were common adverse events, occurring in 5 of 28 patients (17.9%) [11]. Psychiatric adverse effects such as irritability and aggression have been reported with PER [37]. However, no such symptoms were observed in the present cohort. These findings may reflect the relatively low PER doses and suggest favorable tolerability of PER monotherapy.

Given this safety profile and current SNO/EANO recommendations against routine long‑term ASM [9], routine postoperative ASM may not be necessary in low–risk patients. Among the 22 patients without early postoperative seizures, ASM was discontinued in 9 patients within 1–6 months, and none experienced subsequent seizures. In glioma-related epilepsy, tumor progression is closely associated with increased seizure frequency, particularly in high-grade gliomas, where seizure recurrence often occurs concurrently with tumor regrowth or recurrence [38]. Therefore, routine postoperative ASM may not be necessary in patients at low risk of epilepsy, but careful clinical and radiological follow-up remains essential to anticipate seizure recurrence.

### Limitations of the study

This study has several limitations. First, the sample size was small and the study was conducted at a single institution, which limits the statistical power and generalizability. In addition, PER dosing, timing of initiation, and postoperative ASM tapering were based on retrospective observational data. In particular, the dosing level was determined by the treating physicians, and patients with higher epileptogenic risk tended to receive the 4 mg dose, which may have introduced selection bias. Moreover, given that adverse events were evaluated through routine clinical observation rather than structured assessments and that patients undergoing AC for gliomas often exhibit baseline neuropsychiatric symptoms, some neuropsychiatric changes may not have been fully detected.

Second, the perioperative ASM regimen in this study included both oral and intraoperative intravenous PER dose, which makes it difficult to determine the contribution of each component. On the day of surgery, seizure control may have been achieved with intraoperative intravenous administration alone, without postoperative dosing. This possibility is supported by the pharmacokinetic profile of PER, which has a long elimination half–life of approximately 105 h [34, 35]. 

Finally, the follow‑up period was relatively short, with most patients observed for up to 15 months, so long‑term seizure control and late adverse events were not fully evaluated. Future large‑scale, prospective multicenter studies with standardized perioperative protocols and longer follow‑up should be conducted.

## Conclusions

PER provided perioperative seizure control with a very low incidence of adverse effects in this cohort. These exploratory results support the feasibility of PER monotherapy as a perioperative antiseizure strategy during AC and highlight the need for prospective studies to further assess its prophylactic efficacy and optimal dosing.

## Data Availability

The datasets generated and analysed during the current study are not publicly available due to patient privacy and institutional regulations but are available from the corresponding author on reasonable request and with approval from the institutional review board.
